# Genetic Assessment of African Swine Fever Isolates Involved in Outbreaks in the Democratic Republic of Congo between 2005 and 2012 Reveals Co-Circulation of p72 Genotypes I, IX and XIV, Including 19 Variants

**DOI:** 10.3390/v9020031

**Published:** 2017-02-18

**Authors:** Leopold K. Mulumba–Mfumu, Jenna E. Achenbach, Matthew R. Mauldin, Linda K. Dixon, Curé Georges Tshilenge, Etienne Thiry, Noelia Moreno, Esther Blanco, Claude Saegerman, Charles E. Lamien, Adama Diallo

**Affiliations:** 1 Central Veterinary Laboratory, Avenue Wangata, P.O. Box 8842, Kinshasa I, Democratic Republic of Congo; labovetkin@yahoo.fr (L.K.M.-M.); george.tshilenge@sacids.org (C.G.T.); 2 Research Unit of Epidemiology and Risk Analysis Applied to Veterinary (UREAR-Ulg), Fundamental and Applied Research for Animals & Health, Faculty of Veterinary Medicine, University of Liege, 4000 Liege, Belgium; etienne.thiry@ulg.ac.be (E.T.); claude.saegerman@ulg.ac.be (C.S.); 3 Animal Production and Health Laboratory, International Atomic Energy Agency, Wagramer Strasse 5, P.O. Box 100, A-1400 Vienna, Austria; c.lamien@iaea.org (C.E.L.); adama.diallo@cirad.fr (A.D.); 4 Centers for Disease Control and Prevention, Atlanta, GA 30333, USA; MMauldin@cdc.gov; 5 Pirbright Institute, Ash Road, Pirbright, Woking, Surrey GU24 ONF, UK; linda.dixon@pirbright.ac.uk; 6 Centro de Investigaciόn en Sanidad Animal (CISA), INIA, Valdeolmos, 28130 Madrid, Spain; moreno.noelia@inia.es (N.M.); blanco@inia.es (E.B.); 7 Oak Ridge Institute for Science and Education (ORISE) CDC Fellowship Program, Oak Ridge, TN 37830, USA

**Keywords:** African swine fever virus, outbreaks, Democratic Republic of Congo, swine, genotypes, molecular epidemiology, *p72* gene, *p54* gene, CVR

## Abstract

African swine fever (ASF) is a devastating disease of domestic pigs. It is a socioeconomically important disease, initially described from Kenya, but subsequently reported in most Sub-Saharan countries. ASF spread to Europe, South America and the Caribbean through multiple introductions which were initially eradicated—except for Sardinia—followed by re‑introduction into Europe in 2007. In this study of ASF within the Democratic Republic of the Congo, 62 domestic pig samples, collected between 2005–2012, were examined for viral DNA and sequencing at multiple loci: C-terminus of the *B646L* gene (p72 protein), central hypervariable region (CVR) of the *B602L* gene, and the *E183L* gene (p54 protein). Phylogenetic analyses identified three circulating genotypes: I (64.5% of samples), IX (32.3%), and XIV (3.2%). This is the first evidence of genotypes IX and XIV within this country. Examination of the CVR revealed high levels of intra-genotypic variation, with 19 identified variants.

## 1. Introduction

African swine fever (ASF) is a complex and highly lethal haemorrhagic disease of domestic swine with mortality rates reaching 100%. ASF, which is threatening the world pig industry, is a notifiable disease by the World Organization for Animal Health (OIE) [1]. Since the recognition of ASF in Kenya in the 1920s [2], ASF has expanded to most sub-Saharan countries [3], and was exported outside Africa in 1957 to Portugal [4]. Subsequent exportations to Europe occurred in 1960 and 2007 [5,6]. Introduction of ASF has not been limited to Europe, as outbreaks with putative causal links to Spain have occurred in the Caribbean and South America [7].

Domestic pigs are most susceptible, with the disease course ranging from peracute, acute, subacute, chronic and unapparent and, mortality rates ranging from 100% to as little as 3% [8]. ASF is caused by African swine fever virus (ASFV), which is transmitted to swine through three main routes: (1) a sylvatic cycle involving wild swine and *Ornithodoros* ticks; (2) from the sylvatic cycle to domestic pigs; and (3) domestic pig cycle involving domesticated pig to pig transmission [3,9]. ASFV is a large arbovirus within the genus *Asfivirus* and is the sole member of the family *Asfarviridae* [10].

Due to the lack of treatment and vaccine, rapid and accurate diagnosis complemented by the genotyping of circulating ASFVs may contribute to timely improvement of prevention and control strategies. In order to identify and determine the heterogeneity of circulating ASFVs, a rapid method of polymerase chain reaction (PCR)-based sequencing of a 478 base pair (bp) fragment at the C-terminal end of the *p72* gene has been commonly used to differentiate the different genotypes of ASFV [11]. Given the low level of genetic variation detected at the *p72* locus among ASFVs recovered in domestic pig outbreaks, examination of more variable genomic regions such as the central hypervariable region (CVR) of the *B602L* gene [12] in combination with the *p54* and *p30* genes [13] were used to differentiate between isolates within a single outbreak.

Currently, there are 23 confirmed genotypes of ASFV based on the sequencing of the *p72* gene [11], not all of which are known to be currently circulating. The most recent, genotype XXIII, was discovered in Ethiopia [14]. In Africa, further diagnostic analysis of suspected outbreaks shows genotype I continuing to circulate in Western and Central Africa [15,16]. Countries with continued presence of genotypes IX and X include Uganda and Kenya [17,18,19]. Genotype II has maintained its presence in Tanzania, Mozambique, Madagascar and Zambia, and is what led to the introduction of genotype II into Georgia in the Caucasus region in 2007 [6,20]. Since that time, ASFV has spread from Georgia and the Caucasus to the Baltic states (Estonia, Latvia and Lithuania), the Russian Federation, Ukraine, and Poland [21,22,23].

The Democratic Republic of the Congo (DRC) is the second largest country in Africa, with the central and western portions of the country being dominated by the second largest block of rainforest in the world, whereas the southern and eastern regions are characterized by savannas. The 9000-km perimeter of the DRC contacts nine countries and includes a number of lakes (e.g., Edward, Albert, Kivu, Tanganyika) and rivers (e.g., the Congo, Ubangi, Kasai, Semliki). These geographic elements, as well the presence of many trans-frontier tribes that inhabit the DRC and surrounding countries contribute to the semi-porous nature of the DRC’s political boundary.

In 2011, the DRC reported 84 outbreaks and a loss of 105,614 swine, leading African countries in both statistics [24]. Despite the prevalence of ASF in the DRC, the variety of genotypes reported in surrounding countries [9,19], and the large number of studies that have examined this disease, no in-depth study has focused on understanding the genetic diversity of ASFV within the DRC. The goal of this study was to improve the scientific community’s understanding of ASFV strains circulating in the DRC. To achieve this goal, we collected samples from swine that exhibited ASF clinical signs and pathological findings over a broad geographic and temporal range. We utilized a multi-locus genotyping approach to categorize gene sequences into genotypes, and used this data to improve the understanding of the natural history as well as the links between outbreaks of ASFV in the DRC.

## 2. Materials and Methods

### 2.1. Study Area and Samples

A total of 62 tissue samples (spleen, lymph node, kidney, lung, liver, heart and stomach) of ASF suspected cases were collected between 2005 and 2012 from 57 domestic pig carcasses and from 25 locations. Of these carcasses, 54 were collected in outbreaks and three were slaughtered in urban markets. Sampling localities were located in six provinces (Kinshasa, Equateur, Katanga, Orientale, Bas-Congo and Maniema) that contain the majority of the country’s domestic pig population. Tissue samples were transported to the laboratory, homogenized and supernatants were stored at −80 °C until use. Samples, collection localities, and other details are provided in Table 1.

### 2.2. African Swine Fever (ASFV) DNA by Polymerase Chain Reaction (PCR)

ASFV DNA was extracted from tissue samples using the QIAGEN blood and tissue extraction kit (Qiagen, Hilden, Germany) according to the manufacturers’ protocol. Each sample of extracted DNA was then tested by real-time PCR (qPCR), as described by King et al. [25] to confirm the presence of viral DNA for ASFV using the primers King forward (5′-CTGCTCATGGTATCAATCTTATCGA-3′), King reverse (5′-GATACCACAAGATCRGCCGT-3′), and the King probe (5′‑Fam‑CCACGGGAGGAATACCAACCCAGTG-Tam-3′).

### 2.3. Generation of ASFV Sequence Data

Samples that were positive by qPCR had each target gene fragments amplified separately using the PCR protocols outlined below. The C terminal end of the *B646L* (*p72*) gene was amplified using primers p72U (5′-GGCACAAGTTCGGACATGT-3′) and p72D (5′‑GTACTGTAACGCAGCACAG-3′) as recommended [11]. The CVR locus was amplified using primers, ORF9RLW_F (5′-AATGCGCTCAGGATCTGTTAAATCGG-3′) and ORF9RLW_R (5′‑TCTTCATGCTCAAAGTGCGTATACCT-3′) as described [26], The full *E183L* (*p54*) gene was amplified using primers P54F (5′-GCCTGCGGATTCTGAAGATA-3′), and P54R (5′‑AGGACGCAATTGCTTAAACG-3′) using a touchdown PCR protocol as follows, 95 °C for 5 min, followed by 15 cycles of 95 °C, 30 s, 60 °C, 30 s, 72 °C, 1 min, then 25 cycles of 95 °C, 30 s, 58 °C, 30 s, 72 °C, 1 min, with a final extension or 72 °C for 5 min. PCR products were purified using Wizard SV Gel and PCR Clean Up kit, according to the manufacturers’ protocol (Promega Corporation, Madison, WI, USA). Purified PCR products were submitted to LGC Genomics (Berlin, Germany) with amplification primers, for sequencing. Raw sequences were assembled and edited using Vector NTI 11.5 Software (Life Technologies, Carlsbad, CA, USA). Sequences were then aligned with GenBank reference sequences using MEGA (Version 6.0) or BioEdit (Version 7.2.3) using the ClustalW method. All nucleotide sequences were deposited in GenBank (Accession # KX121429‑KX121600).

### 2.4. Molecular Characterization of ASFV

Multiple sequence alignments of both the *p54* and *p72* genes were generated in MEGA (Version 6.0) [27] using default values of the ‘by codon’ option with the ClustalW algorithm with additional manual editing as needed. The *p72* alignment was 404 bp in length and contained 120 sequences. Of these 120 sequences, 62 were generated for this study and 58 were reference sequences with at least one representing each of the known 23 genotypes. The *p54* alignment was 657 bp in length and included 84 sequences; 34 were generated for this study, and 50 were reference sequences for 20 of the 23 known *p72* genotypes. Published sequences for genotypes XI, XII, and XVIII were unavailable for examination. The most appropriate model of molecular evolution was determined by the corrected Akaike Information Criterion (AICc) using MEGA [27]. Maximum likelihood (ML) analyses with 1000 bootstrap replicates were performed using the program MEGA with the predetermined model of molecular evolution (GTR+I+G for the *p72* dataset and HKY+G for the *p54* dataset) using all sites. The intra-genotypic genetic distances for the *p72* and *p54* nucleotides sequences of DRC samples were calculated using MEGA [27].

### 2.5. Central Hypervariable Region (CVR) of *B602L* Gene

Grouping of amino acids into tetramers at this locus has been utilized by other researchers, therefore the coding of tetramers followed methods outlined previously [20,26,28,29,30]. The amino acid tetramer codes are provided in Table 2.

## 3. Results

### 3.1. Clinical Findings and African Swine Fever (ASF) Diagnosis

#### Field Identification and Description of Collecting Localities

Fifty-four out of the 57 sampled pigs exhibited many of the following signs or pathological findings: hemorrhagic edema; enlargement of spleen and some internal lymph nodes; hydropericardium and pericarditis; hydrothorax; ascites; as well as skin cyanosis and petechiae. The three remaining pigs sampled in the markets appeared superficially healthy, but presented with enlarged, congestive or hemorrhagic spleens and/or gastrohepatic lymph nodes. Massive mortality was documented in the 25 sampled localities. Four of the 25 sampling sites, including the cities of Yakoma, Boende, Mahagi and Kipushi, recorded indigenous pigs of local breeds, primarily free ranging to be the most commonly lost. The remaining 21 locations were commercial farms raising improved breeds of pigs in backyards or securely fenced areas, minimizing intermingling of wildlife and domestic swine.

### 3.2. Laboratory Diagnostics

Real time PCR identified 62 samples (100%) as positive for ASFV DNA and distributed per location as follows: Ngafula (*n* = 17) Boende (*n* = 11), Ngaliema (*n* = 8), Nsele (*n* = 7) Yakoma (*n* = 5) Limete (*n* = 4), Mayanda (*n* = 3), Mahagi (*n* = 2), Kasavubu (*n* = 1), Kintambo (*n* = 1), Kipushi (*n* = 1), Lingwala (*n* = 1), Maniema (*n* = 1) and Ndjili (*n* = 1). The *p72* gene was sequenced for all 62 positive samples and 55 positive samples were sequenced for the *p54* gene and 54 for the CVR (Table 1).

### 3.3. Molecular Characterization of ASFV

**Figure 1:**
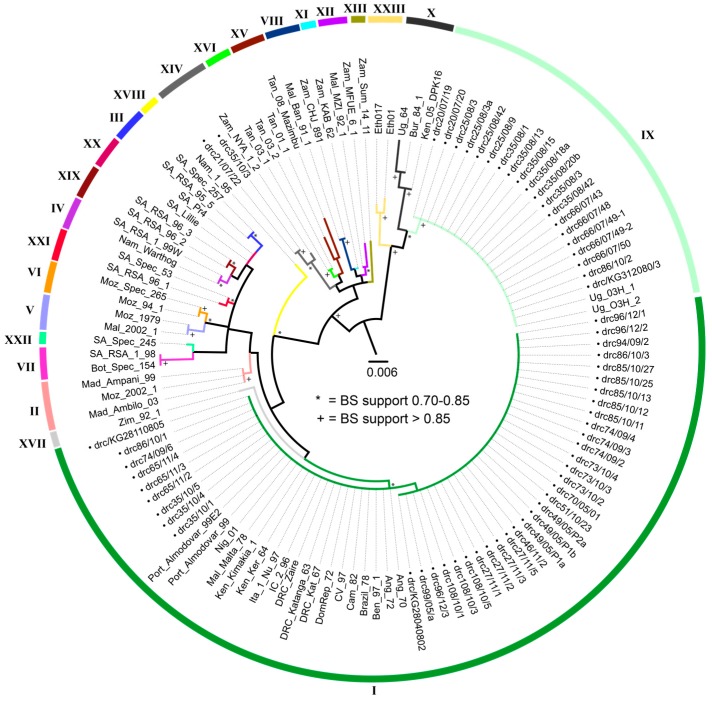
Maximum likelihood tree depicting genetic relationships of strains examined in this study utilizing the *p72* locus. Bootstrap support (BS) is indicated by * for values between 75% and 85%, and by + for values greater than 85% support. The scale bar indicates the number of nucleotide substitutions per site. Each color is a different genotype, indicated by the corresponding roman numerals. Labels for all strains generated by this study begin with •drc (Democratic Republic of the Congo). Strains are named in the following manner: Country_Strain Name. Acronyms used for countries of origin are as follows: Ang = Angola, Ben = Benin, Bot = Botswana, Bra = Brazil, Bur = Burundi, Cam = Cameroon, CV = Cape Verde, Dom Rep = Dominican Republic, the DRC = Democratic Republic of Congo, IC = Ivory Coast, Ita = Italy, Ken = Kenya, Mad = Madagascar, Mal = Malawi, Malt = Malta, Moz = Mozambique, Nam = Namibia, Nig = Nigeria, Port = Portugal, SA = South Africa, Tan = Tanzania, Ug = Uganda, Zam = Zambia, Zimb = Zimbabwe.

Phylogenetic analyses of the *p72* gene revealed that the newly sequenced ASFV strains which circulated in the DRC from 2005 to 2012 clustered into three *p72* genotypes: I, IX and XIV (Figure 1). Of the newly analyzed stains, 40 (64.5%) grouped with strains previously identified as belonging to genotype I, including three published strains from the DRC (Katanga63 (Genbank: AF301540) [11], Kat67 (Genbank: FJ174377) [13] and Zaire (Genbank: AY351515) [9]); 20 strains (32.3%) were recognized as genotype IX, and 2 (3.2%) belong to genotype XIV. This is the first report of genotypes IX and XIV circulating in the DRC. Although sequences generated herein grouped with high bootstrap support (>75%) with reference samples of their respective genotypes, support for both inter- and intra-genotypic relationships varied.

**Figure 2:**
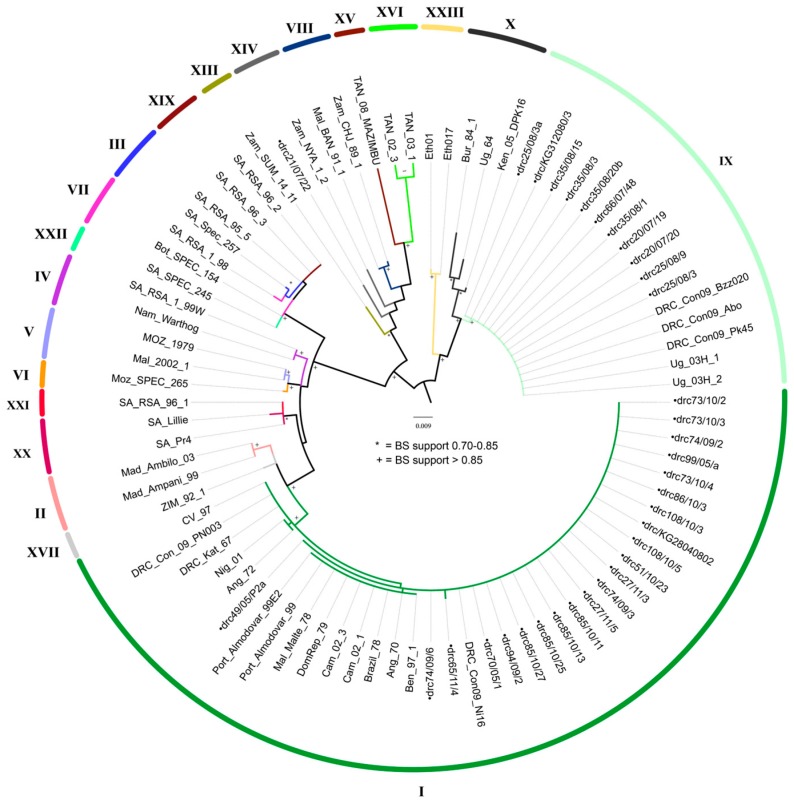
Maximum likelihood tree depicting genetic relationships of examined strains using the *p54* locus (*E183L* gene). Bootstrap support values greater than 75% are shown. The scale bar indicates the number of nucleotide substitutions per site. The colored clades indicate genotypes represented by strains sequenced in this study. Labels for all strains generated by this study begin with •drc. Strains are named in the following manner: Country_Strain Name. Acronyms used for countries of origin are as follows: Ang = Angola, Ben = Benin, Bot = Botswana, Bra = Brazil, Bur = Burundi, Cam = Cameroon, CV = Cape Verde, Dom Rep = Dominican Republic, the DRC = Democratic Republic of Congo, IC = Ivory Coast, Ita = Italy, Ken = Kenya, Mad = Madagascar, Mal = Malawi, Malt = Malta, Moz = Mozambique, Nam = Namibia, Nig = Nigeria, Port = Portugal, RC = Republic of Congo, SA = South Africa, Tan = Tanzania, Ug = Uganda, Zam = Zambia, Zimb=Zimbabwe.

Phylogenetic analyses of the *p*54 gene recovered the same groupings of new sequences with respective genotypes as the *p72* analyses with high bootstrap support (>83%) (Figure 2). The arithmetic means of nucleotide substitutions per site between the DRC ASFVs of each of the three genotypes (within group mean distance) were estimated using MEGA version 6. The within group mean distance for the *p72* nucleotide sequences were 0.002 for genotype I and 0.0 for genotype IX and XIV. Likewise, for the *p54* nucleotide sequences, the within group mean distance was 0.02 for the genotype I members, and 0.0 for genotype IX members. Only one isolate of genotype XIV was successfully amplified and sequenced for this gene.

### 3.4. CVR of *B602L* Gene

**Figure 3:**
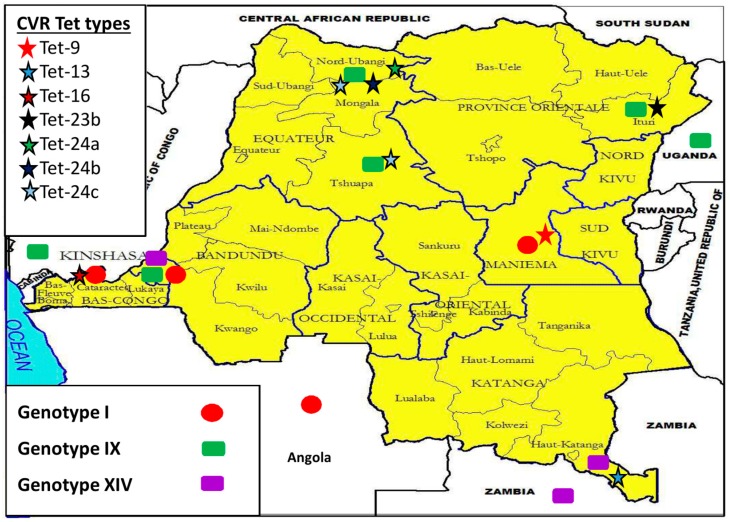
Provincial localization of revealed *p72* genotypes and their corresponding central hypervariable region (CVR) tet-types within the DRC, as well as some historical African swine fever virus (ASFV) genotypes from neighboring countries.

**Figure 4:**
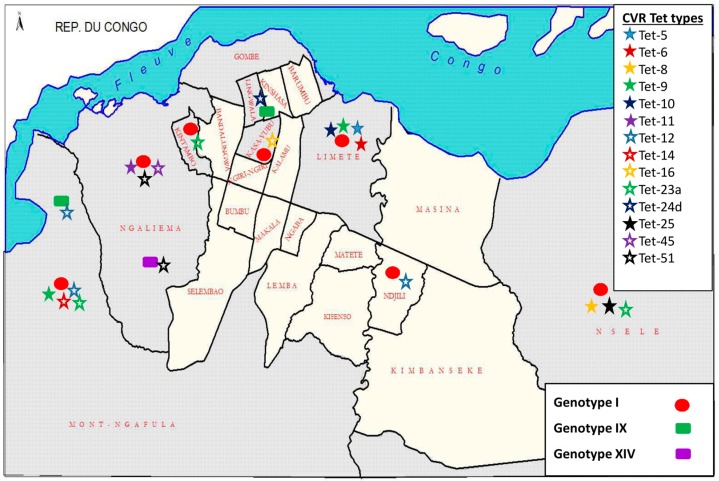
Localization of ASFV *p72* genotypes and the corresponding CVR tet-types in this study within the Kinshasa City Province.

Sequence analysis of the CVR locus showed distinct variability in nucleotide sequence and recognized 19 unique nucleotide sequences, which were translated into amino acid sequences, and subsequently coded as amino acid tetramers (tet-types). Thirteen tet-types were detected within strains identified as belonging to *p72* genotype I, six from strains belonging to genotype IX, and two within genotype XIV strains (Table 2). All strains grouped within a tet-type contained identical CVR nucleotide sequences; therefore, no resolution was lost by converting nucleotide sequences to tetrameric repeat sequences. Tet-12 and 51 were both detected in *p72* genotype I strains, but tet-12 was also found in a *p72* genotype IX strain (drc86/10/2), and tet-51 was also found in a *p72* genotype XIV strain (drc35/10/3). All three *p72* genotypes and 14 tet-types were identified throughout Kinshasa (Table 2, Figure 4). Regarding the five remaining provinces: Equateur presented four tet‑types (within genotype IX); Bas-Congo and Maniema each had a single tet-type (within genotype I); Katanga (within genotype XIV) and Oriental each contained a single tet‑type (within genotype IX), as well (Figure 3).

### 3.5. Democratic Republic of the Congo (DRC) ASFV Genotypes Geographical Distribution

Different ASFV strains were identified in the provinces of Bas-Congo, Equateur, Katanga, Kinshasa, Maniema, and Orientale (Figure 3). Genotype I strains were detected in Bas-Congo (Localities-Mayanda), Kinshasa (Localities-Limete, Ngafula, Ngaliema, Kasavubu, Nsele, Kintambo, Ndjili, Nsele, Kinshasa) (Figure 4) and Maniema (Locality-Maniema), whereas genotype IX strains were recovered from Equateur (Localities-Boende, Yakuma), Kinshasa (Localities-Ngafula, Lingwala), and Oriental provinces (Locality-Mahagi). Genotype XIV was detected from Katanga (Locality-Kipushi) and Kinshasa (Locality-Ngaliema).

## 4. Discussion

### 4.1. Molecular Characterization

**Figure 5:**
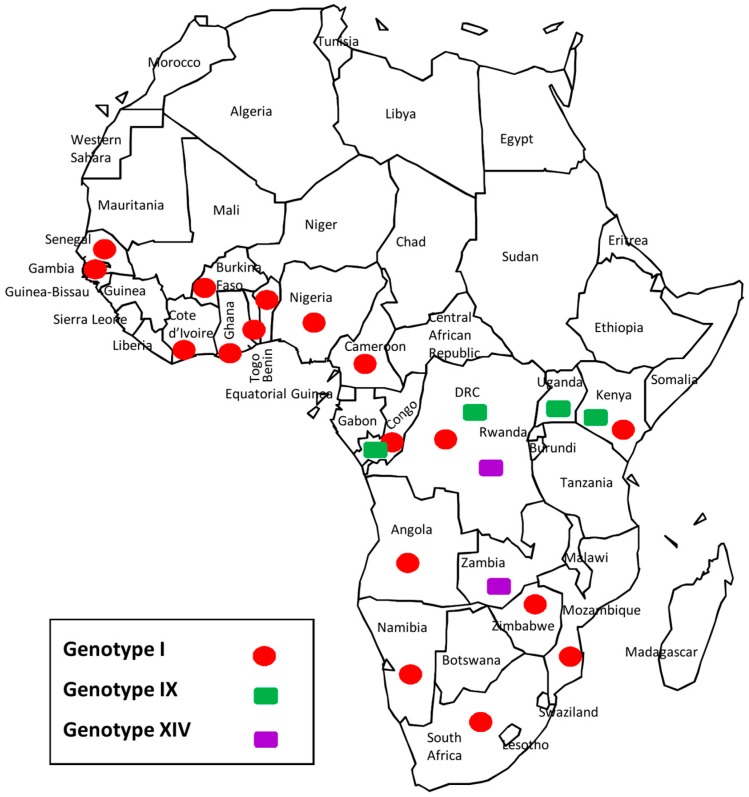
Continental distribution of the three *p72* genotypes revealed by this study in the DRC. Position of symbols represents the presence of a genotype within the country, and is not indicative of specific geographic localities.

This is the first extensive molecular evaluation of circulating ASFV genotypes in the DRC. Previous knowledge was based on data from single samples submitted for ASF diagnosis [9,11,26,29,31,32], but little was known about the dynamics of circulating ASFV strains in the DRC. Analysis of the *p72* gene identified three genotypes (I, IX and XIV) circulating in the DRC. Forty of 62 (64.5%) clustered with DRC historical strains (DRC_Kat63 and DRC_Kat67), as well as other strains from genotype I, confirming that genotype I is the most prevalent in the country. Additionally, DRC strains of this genotype exhibited limited genetic variability, which has been repeatedly documented and hypothesized to be predominantly a result of maintenance through the domestic pig cycle [9]. There were 19 *p72* genotype IX strains (30.6%) with no genetic variation detected. Previously undocumented within the DRC, genotype IX is much more geographically restricted, as it is endemic to East and Central Africa (Figure 5), having been reported previously in Republic of Congo, Uganda, and Kenya (Figure 5) where it is involved in sylvatic and domestic cycles [9,17]. Genotype XIV was also not previously reported in the country prior to the two (3.2%) strains reported herein. Genotype XIV was previously only reported from Zambia (Figure 5), where it was originally isolated from a tick of the genus *Ornithodoros* in 1986 [9]. In regards to the distribution of ASFV within the DRC recovered in this study, all genotypes were found near the eastern and western borders, and confirmed localities for all three genotypes occurred in the southern half of the country; however, only genotype IX viruses were collected from localities in the northern DRC (Figure 3 and Figure 4). The presence of all three genotypes within the Kinshasa province is likely a result of pig shipments, as pork sold at markets in Kinshasa has been documented to originate from multiple regions of the country, including the provinces of Equateur, Bandundu, and Bas-Congo [33].

Although the *p54* gene was previously determined to be a valuable locus for finer levels of discrimination [19,34,35], no additional resolution was gained for this dataset; however, the p54 locus did corroborate the topology generated by the *p72* analysis. Given the sampling scheme of this study (domestic swine only), failure to achieve higher resolution may be due to the low genetic variation consistently detected within the domestic pig cycle, when compared to the higher levels of variation previously detected within the sylvatic cycle [9].

The CVR was capable of providing further resolution than either the *p72* or *p54* genes. The pattern of genetic variability varied dependent upon the *p72* genotype examined. Within *p72* genotype I strains, genetic variation was primarily a result of variability in the number of tetramers as previously reported [36], specifically the tetrameric amino acid repeats CAST and CVST. Also of interest within genotype I, is that tet-25, 45, and 51 (also found in one genotype XIV strain) contained a conserved sequence of 11 tetramers (tetrameric repeat sequence number (TRS) ABNABNBT[D/A]BN) not found in other tetrameric sequences within this genotype. This sequence was flanked by a variable number of ‘A’ coded tetramers. The historical isolate Kat67 genotype I CVR was tet-23, but had a similar sequence BNAxx. Additionally, BTDBN also flanked on each end by variable repeats of ‘A’ [26]. The new genotype XXIII from Ethiopia had one CVR sequence, ETH/3, which also has a similar motif with xxBNABTDBxx [14]. Nigerian sequences are of genotype I and also share part of this motif in the CVR region xxABNABNxx [16] (Table 2). CVR tet‑types of *p72* genotype IX consisted primarily of a more complex sequence than genotype I strains, with the exception of tet-12 (common to genotype I strains) being documented in one genotype IX strain. Interestingly, CVR tet-23b which is present in two recent DRC genotype IX strains from 2007 is identical to two genotype IX CVR sequences from Uganda in 2003 and Kenya in 2006 [13] (Table 2). Of the two tet-types detected in genotype XIV strains, the first (tet-51) was identical to a tetrameric repeat sequence found in a genotype I strain, and the second was extremely different than any other tet-types reported herein (Table 2). Several CVR tet-types are discussed below in reference to the detection of multiple strains within outbreaks, and potential links between outbreaks based on this locus.

### 4.2. Disease Ecology, Molecular Epidemiology and Case Investigations

The higher resolution offered by the CVR allowed for the three genotypes to be further broken down into 19 variants. The high level of variability previously noted in the CVR [9,37,38] would suggest that highly similar/identical CVRs are more closely related than more divergent CVRs; however, given the nature of nucleotide repeat regions, such as the CVR, identical sequences can occur due to homoplasy. With these possibilities in mind, a number of putative outbreak connections are proposed and discussed below, as are details of co-circulation of multiple variants.

The detection of genotype I, tet-23 strains during the 2009 outbreaks at Nsele and Kintambo, as well as the 2011 outbreak in Ngafula may suggest that these geographically proximal outbreaks were caused by closely related strains, even though they occurred over a two-year time span which could be facilitated by the asymptomatic infection of domestic pigs [39] in the area. A second potential connection is suggested by analysis of sequences from the Mahagi outbreak in April 2007 (near the Ugandan border), as they were of identical *p72* genotype IX and tet-23b to strains from Uganda (Ug03H.1, Genbank: GQ916933), and Kenya (Ken06.B1, Genbank: GQ916935 & Ken06.Bus, Genbank: GQ916940) examined previously [13]. A third connection stems from a strain from a clinically healthy pig sampled at the Kinshasa market (drcKG28040802) in 2008. This strain belonged to the same genotype I and tet-16 as samples from the 2012 outbreak in Mayanda, Bas-Congo province. The geographic proximity of these two provinces, in combination with the high level of commercial traffic between them, could easily result in the spread of ASF from one province to the other [1,20].

Again, either the ability of ASFV to persist in asymptomatic pigs (domestic or feral) or the sylvatic cycle (including ticks) could explain how closely related strains were responsible for outbreaks separated by four years, as Mayanda is a rural locality, where there is a high potential for interaction between domestic and feral swine. Another potential outbreak link was made apparent upon examination of genotype IX strains collected from outbreaks in Yakoma (April 2007) and Boende (March and April 2008). Tet-24c strains were detected in both outbreaks, which severely affected both feral and domestic pigs [40]. The Boende outbreak was estimated to have caused more than 4500 swine fatalities [40]. Both Yakoma and Boende are forested sites, located in North Ubangi and Tshuapa Districts of the Equateur Province, respectively. The forest environment and free ranging animal husbandry practices enable interaction between domestic and wild swine, potentially allowing for crossover between infection cycles and the occurrence of multiple strains.

Another instance of variation occurred in the Ngaliema 2010 outbreak, where tet-51 was found in two strains: drc35/10/1 (genotype I) and drc/35/10/3 (genotype XIV). Co-infection of different tet‑types was even documented within the same pig, when two genotype I strains, drc49/05/p1a (tet‑5) and drc49/05/p1b (tet-9), were extracted from the spleen and lymph node respectively, of a pig in 2005 from Kinshasa. In similar studies conducted in Mozambique and Nigeria, individual co‑infection was not observed [38,41].

Understanding the details of ASFV circulation across the DRC is clearly complex. Co-circulation of genotypes and tet-types and co-infection of multiple tet-types within a single pig suggests a high level of genetic variation and potential support of a previous hypothesis of recombination [42,43]. However, detection of conserved tet-types across large geographic areas, and over multiple years suggests that current markers can provide insight into the movement of ASFV strains. Unfortunately, connecting cases is not straight forward, as anthropogenic factors, such as trade of pigs and animal husbandry practices can play a role. Whole genome examination of these strains may provide a more definitive understanding of relationships.

As the DRC is the largest country in SSA, and borders nine countries, understanding the prevalence and distribution of ASFV genotypes within the DRC is an important step in better understanding large scale patterns of ASFV. Given the high degree of similarity, at all examined loci, between genotype IX strains collected in Mahagi, the DRC (presented herein), and Uganda strains of a previous study [13], it appears that the distribution of this strain spans the border between the DRC and Uganda, suggesting that this strain has been transmitted across boundaries by movement of either feral or domesticated swine. Two more potential cross-boundary transmissions of ASF into/out of the DRC are worth mentioning; however, their molecular evidence is less direct. The first possibility is that genotype XIV may have been transferred, between Zambia (Zam_NYA/12-(Genbank: AY351555), isolated in 1986) [9] and the DRC (drc21/07/22, strain from 2007) through Kipushi, as *p72* sequences between these strains share 99.26% nucleotide sequence identity. Unfortunately, no CVR sequence for strain Zam_NYA/12 was available for a more detailed comparison. The next putative trans-boundary migration of ASF could have occurred between Brazzaville, Republic of Congo (Con09/Ni16-(Genbank: HQ645947), isolated 2009) [17] and the DRC (drc86/10/1, strain from in 2010). The *p72* sequences were identical, and the CVR locus differed by the insertion of two amino acid tetramers coded as A (AAAAAAAAAF from the Brazzaville strain and AAAAAAAAAAAF in the DRC strain). Rapid mutation rates have been shown in vitro [44], therefore, given the highly variable nature of the CVR, it is difficult to omit the possible link between these outbreaks, especially as the boundary is a narrow aquatic border with high levels of human and animal traffic.

This first, in-depth examination of ASFV in the DRC, has provided evidence of (1) circulation of multiple genotypes previously not reported within the DRC; (2) putative links between both geographically and temporally separated outbreaks; (3) potential movement of ASFV strains across borders between the DRC and Uganda, Zambia, Congo; (4) co-circulation of multiple ASFV genotypes within outbreaks and (5) a pig co-infected with two tet-types. These data, in combination with examination of genotype relationships, will be useful for the optimization of current prevention and control strategies at the regional level given the location and size of the DRC in relation to the rest of the continent and those countries also dealing with ASF.

## Figures and Tables

**Table 1:** Samples, details, and genotypes.

Virus	Outbreak Date	Location	GPS	Ecological Profile	Tissues	Farming System	Genotype
drc49/05/p1a	May 2005	Limete	4°18 S/15°22 E	City	Spl	Commercial	I
drc49/05p1b	May 2005	Limete	4°18 S/15°22 E	City	Ln	Commercial	I
drc49/05/P2a	May 2005	Limete	4°18 S/15°22 E	City	Spl	Commercial	I
drc70/05/1	2005	Limete	4°18 S/15°22 E	City	Spl	Commercial	I
drc75/05/1	2005	Maniema	2°93 S/25°86 E	City	Spl	Commercial	I
drc99/05/a	2005	Ngafula	4°21 S/15°14 E	Peri-urban	Spl	Commercial	I
drcKG28110805	Nov 2008	Ngaliema	4°21 S/15°21 E	City	Spl	Backyard	I
drcKG28040802	Apr 2008	Kasavubu	4°20 S/15°20 E	City	Spl	Backyard	I
drc74/09/2	2009	Nsele	4°24 S/15°30 E	Peri-urban	Kd	Commercial	I
drc74/09/3	2009	Nsele	4°24 S/15°30 E	Peri-urban	Lg	Commercial	I
drc74/09/4	2009	Nsele	4°24 S/15°30 E	Peri-urban	Lv	Commercial	I
drc74/09/6	2009	Nsele	4°24 S/15°30 E	Peri-urban	Hrt	Commercial	I
drc94/09/2	2009	Kintambo	4°20 S/15°18 E	City	Ln	Commercial	I
drc35/10/1	2010	Ngaliema	4°21 S/15°05 E	City	Spl	Backyard	I
drc35/10/5	2010	Ngaliema	4°21 S/15°05 E	City	Ln	Backyard	I
drc35/10/4	2010	Ngaliema	4°21 S/15°05 E	City	Ln	Backyard	I
drc51/10/23	2010	Ndjili	4°24 S/15°21 E	City	Spl	Commercial	I
drc73/10/2	2010	Ngaliema	4°21 S/15°05 E	City	Kn	Backyard	I
drc73/10/3	2010	Ngaliema	4°21 S/15°05 E	City	Lv	Backyard	I
drc73/10/4	2010	Ngaliema	4°21 S/15°05 E	City	Ln	Backyard	I
drc85/10/13	2010	Ngafula	4°21 S/15°14 E	Peri-urban	Spl	Commercial	I
drc85/10/12	2010	Ngafula	4°21 S/15°14 E	Peri-urban	Hrt	Commercial	I
drc85/10/11	2010	Ngafula	4°21 S/15°14 E	Peri-urban	Lg	Commercial	I
drc85/10/27	2010	Ngafula	4°21 S/15°14 E	Peri-urban	Hrt	Commercial	I
drc85/10/25	2010	Ngafula	4°21 S/15°14 E	Peri-urban	Lv	Commercial	I
drc86/10/1	2010	Ngafula	4°21 S/15°14 E	Peri-urban	Kd	Commercial	I
drc86/10/3	2010	Ngafula	4°21 S/15°14 E	Peri-urban	Lv	Commercial	I
drc108/10/3	2010	Ngafula	4°21 S/15°14 E	Peri-urban	Lv	Commercial	I
drc108/10/5	2010	Ngafula	4°21 S/15°14 E	Peri-urban	Spl	Commercial	I
drc27/11/1	2011	Ngafula	4°21 S/15°14 E	Peri-urban	Spl	Commercial	I
drc27/11/2	2011	Ngafula	4°21 S/15°14 E	Peri-urban	Ln	Commercial	I
drc27/11/3	2011	Ngafula	4°21 S/15°14 E	Peri-urban	Stm	Commercial	I
drc27/11/5	2011	Ngafula	4°21 S/15°14 E	Peri-urban	Lv	Commercial	I
drc65/11/2	2011	Nsele	4°24 S/15°30 E	Peri-urban	Kd	Commercial	I
drc65/11/3	2011	Nsele	4°24 S/15°30 E	Peri-urban	Spl	Commercial	I
drc65/11/4	2011	Nsele	4°24 S/15°30 E	Peri-urban	Lg	Commercial	I
drc96/12/1	2012	Mayanda	5°12 S/15°14 E	Rural	Lg	Village	I
drc96/12/2	2012	Mayanda	5°12 S/15°14 E	Rural	Spl	Village	I
drc96/12/3	2012	Mayanda	5°12 S/15°14 E	Rural	Hrt	Village	I
drc108/10/1	Dec 2010	Ngafula	4°21 S/15°14 E	Peri-urban	Lg	Commercial	I
drc46/11/2	Jun 2011	Kinshasa	4°20 S/15°18 E	City	Hrt	Backyard	I
drc20/07/19	Apr 2007	Mahagi *	2° S/31° E	Rift Valley	Kd	Backyard	IX
drc20/07/20	Apr 2007	Mahagi *	2° S/31° E	Rift Valley	Ln	Backyard	IX
drc25/08/3a	Mar 2008	Boende	0°15 S/21°01 E	Forest	Kd	Free range	IX
drc25/08/3	Mar 2008	Boende	0°15 S/21°01 E	Forest	Spl	Free range	IX
drc25/08/42	Mar 2008	Boende	0°15 S/21°01 E	Forest	Kd	Free range	IX
drc25/08/9	Mar 2008	Boende	0°15 S/21°01 E	Forest	Spl	Free range	IX
drc35/08/1	Apr 2008	Boende	0°15 S/21°01 E	Forest	Spl	Free range	IX
drc35/08/13	Apr 2008	Boende	0°15 S/21°01 E	Forest	Spl	Free range	IX
drc35/08/P4_2_	Apr 2008	Boende	0°15 S/21°01 E	Forest	Spl	Free range	IX
drc35/08/15	Apr 2008	Boende	0°15 S/21°01 E	Forest	Spl	Free range	IX
drc35/08/18	Apr 2008	Boende	0°15 S/21°01 E	Forest	Kd	Free range	IX
drc35/08/20	Apr 2008	Boende	0°15 S/21°01 E	Forest	Spl	Free range	IX
drc35/08/3	Apr 2008	Boende	0°15 S/21°01 E	Forest	Spl	Free range	IX
drc66/07/43	Nov 2007	Yakoma	4° S/22° E	Forest	Lg	Free range	IX
drc66/07/48	Nov 2007	Yakoma	4° S/22° E	Forest	Spl	Free range	IX
drc66/07/49_1_	Nov 2007	Yakoma	4° S/22° E	Forest	Spl	Free range	IX
drc66/07/49_2_	Nov 2007	Yakoma	4° S/22° E	Forest	Ln	Free range	IX
drc66/07/50	Nov 2007	Yakoma	4° S/22° E	Forest	Spl	Free range	IX
drcKG31208/3	Dec 2008	Lingwala	4°20 S/15°19 E	City	Spl	Backyard	IX
drc35/10/3	Apr 2010	Ngaliema	4°21 S/15°05 E	City	Kd	Backyard	XIV
drc21/07/22	2007	Kipushi ^†^	12° S/28° E	City	Spl	Backyard	XIV

drc, Democratic Republic of the Congo; Ln, lymph node; Hrt, heart; Spl, spleen; Kd, kidney; Lg, lung; Lv, liver; Stm, stomach; GPS, global positioning system; *, Uganda border; ^†^, Zambia border.

**Table 2:** Central hypervariable region (CVR) locus-based intra-genotype resolution.

Strain	Location	Year	Genotype	Tetrameric Repeats	TRS
drc35/10/1	Ngaliema *	2010	I	AAAAAAAAAAAAAAAAAAAAAAAAAAAAABNAB NBTDBNAAAAAAAAAAAF	51
drcKG28110805	Ngaliema *	2010	I	AAAAAAAAABNABNBTABNAAAAAAAAAAAAAAAAA AAAAAAAAF	45
drc65/11/4	Nsele *	2011	I	AAAAAABNABNBTDBNAAAAAAAAF	25
drc74/09/2	Nsele *	2009	I	AAAAAAAAAAAAAAAAAAAAAAF	23a
drc74/09/3	Nsele *	2009	I	AAAAAAAAAAAAAAAAAAAAAAF	23a
drc74/09/4	Nsele *	2009	I	AAAAAAAAAAAAAAAAAAAAAAF	23a
drc74/09/6	Nsele *	2009	I	AAAAAAAAAAAAAAAAAAAAAAF	23a
drc94/09/2	Kintambo *	2009	I	AAAAAAAAAAAAAAAAAAAAAAF	23a
drc27/11/3	Ngafula *	2011	I	AAAAAAAAAAAAAAAAAAAAAAF	23a
drc27/11/5	Ngafula *	2011	I	AAAAAAAAAAAAAAAAAAAAAAF	23a
drcKG28040802	Kasavubu *	2008	I	AAAAAAAAAAAAAAAF	16
drc96/12/1	Mayanda	2012	I	AAAAAAAAAAAAAAAF	16
drc96/12/2	Mayanda	2012	I	AAAAAAAAAAAAAAAF	16
drc96/12/3	Mayanda	2012	I	AAAAAAAAAAAAAAAF	16
drc99/05a	Ngafula *	2005	I	AAAAAAAAAAAAAF	14
drc51/10/23	Ndjili *	2010	I	AAAAAAAAAAAF	12
drc85/10/13	Ngafula *	2010	I	AAAAAAAAAAAF	12
drc85/10/27	Ngafula *	2010	I	AAAAAAAAAAAF	12
drc86/10/3	Ngafula *	2010	I	AAAAAAAAAAAF	12
drc86/10/1	Ngafula *	2010	I	AAAAAAAAAAAF	12
drc108/10/1	Ngafula *	2010	I	AAAAAAAAAAAF	12
drc108/10/3	Ngafula *	2010	I	AAAAAAAAAAAF	12
drc108/10/5	Ngafula *	2010	I	AAAAAAAAAAAF	12
drc85/10/12	Ngafula *	2010	I	AAAAAAAAAAF	11
drc73/10/2	Ngaliema *	2010	I	AAAAAAAAAAF	11
drc73/10/3	Ngaliema *	2010	I	AAAAAAAAAAF	11
drc73/10/4	Ngaliema *	2010	I	AAAAAAAAAAF	11
drc49/05/P2a	Limete *	2005	I	AAAAAAAAAF	10
drc85/10/25	Ngafula *	2010	I	AAAAAAAAF	9
drc75/05/1	Maniema	2005	I	AAAAAAAAF	9
drc49/05/p1b	Limete *	2005	I	AAAAAAAAF	9
drc65/11/3	Nsele *	2011	I	AAAAAAAF	8
drc49/05/p1a	Limete *	2005	I	AAAAAF	6
drc70/05/1	Limete *	2005	I	AAAAF	5
Con09/Ni16	Congo ^1^	2009	I	AAAAAAAAAF	10
Kat67	DRC(Zaire) ^2^	1967	I	AAAAAAAABNABTDBNAAAAAAA	23
Nig13_KAF_14	Nigeria ^3^	2014	I	ABNABNAAAAACBNAFA	17
drc66/07/49_1_	Yakoma	2007	IX	AAABBAABBNABBAABBNABNABA	24a
drc66/07/43	Yakoma	2007	IX	AAABNABBBNABBAABBNABNABA	24b
drc66/07/50	Yakoma	2007	IX	AAABNABBBNABBAABBNABNABA	24b
drc66/07/492	Yakoma	2007	IX	AAABNABBBNABBAABBNABNABA	24b
drc66/07/48	Yakoma	2007	IX	AAAABNABBNABBAABBNABNABA	24c
drc35/08/p4_2_	Boende	2008	IX	AAAABNABBNABBAABBNABNABA	24c
drc35/08/18	Boende	2008	IX	AAAABNABBNABBAABBNABNABA	24c
drc35/08/13	Boende	2008	IX	AAAABNABBNABBAABBNABNABA	24c
drc35/08/3	Boende	2008	IX	AAAABNABBNABBAABBNABNABA	24c
drc35/08/20	Boende	2008	IX	AAAABNABBNABBAABBNABNABA	24c
drc35/08/1	Boende	2008	IX	AAAABNABBNABBAABBNABNABA	24c
drc35/08/15	Boende	2008	IX	AAAABNABBNABBAABBNABNABA	24c
drc35/08/3a	Boende	2008	IX	AAAABNABBNABBAABBNABNABA	24c
drc25/08/3	Boende	2008	IX	AAAABNABBNABBAABBNABNABA	24c
drc25/08/9	Boende	2008	IX	AAAABNABBNABBAABBNABNABA	24c
drc25/08/42	Boende	2008	IX	AAAABNABBNABBAABBNABNABA	24c
drcKG31208/3	Lingwala *	2008	IX	AAAABNABBNABBAAABNABNABA	24d
drc20/07/19	Mahagi	2007	IX	AAABNABBNABBAABBNABNABA	23b
drc20/07/20	Mahagi	2007	IX	AAABNABBNABBAABBNABNABA	23b
drc86/10/2	Ngafula *	2010	IX	AAAAAAAAAAAF	12
UG03H.1	Uganda ^4^	2003	IX	AAABNABBNABBAABBNABNABA	23b
Ken06.B1	Kenya ^5^	2006	IX	AAABNABBNABBAABBNABNABA	23b
drc35/10/3	Ngaliema *	2010	XIV	AAAAAAAAAAAAAAAAAAAAAAAAAAAAABNABNBTDBN AAAAAAAAAAAF	51
drc21/07/p22	Kipushi	2007	XIV	AVVOVAVVNBVOV	13
ETH/3	Ethiopia ^6^	2011	XXIII	ABNAAAAACBNABTDBNAFA	20

Codes as labeled in previous studies: [20,26,28,29,30]. TRS, tetrameric repeat sequence number; *, indicates strains collected within Kinshasa Province. A=CAST, CVST, CTST, or CASI; B=CADT, CADI, CTDT, or CAGT; C = GAST or GANT; F = CANT or CAAT; N = NVDT, NVGT, NVDI, or NCDT; T = NVNT; H = RAST; S = SAST; O = NANI, NADI, or NASI; V = NAST, NAVT, NADT, or NANT; D = CASM; G = CTNT; M = NEDT; W = SADT or SVDT; U = NIDT or NTD. Additional sequences utilized in this table from previous studies: ^1^ [17]; ^2^ [26]; ^3^ [16]; ^4^ [13]; ^5^ [13]; ^6^ [14].
